# The Central Nervous System Effects and Mimicry of Common Variable Immunodeficiency (CVID): A Case Report with Literature Review

**DOI:** 10.1155/2019/7623643

**Published:** 2019-09-29

**Authors:** Sohail Farshad, Fernando Figueroa Rodriguez, Alexandra Halalau, Joseph Skender, Cory Rasmussen, Martin Pevzner

**Affiliations:** ^1^Internal Medicine, Beaumont Hospital, Royal Oak 48073, MI, USA; ^2^Rheumatology, Beaumont Hospital, Royal Oak 48073, MI, USA

## Abstract

There is a scarceness of information on the central nervous system effects of common variable immunodeficiency (CVID). A 30-year-old woman with a history of recurrent upper respiratory infections, vitiligo, and immune thrombocytopenic purpura presented with right-sided numbness. Magnetic resonance imaging (MRI) of the thoracic spine revealed a signal hyperintensity. MRI of the brain demonstrated FLAIR hyperintensity in the right middle frontal gyrus. Cerebral spinal fluid was unremarkable. Serum immunoglobulins revealed hypogammaglobulinemia. Endobronchial and subsequent mediastinum biopsies were all negative for sarcoidosis and malignancy. No infectious etiology was found. She was treated with glucocorticoids and intravenous immunoglobulin (IVIG) replacement therapy for CVID-associated myelitis. Follow-up MRI showed improvement; however, her numbness persisted despite these treatments, which led to an outside physician adding methotrexate for their suspicion of sarcoidosis. Her symptoms remained stable for two years, but when the methotrexate dose was weaned, her numbness worsened. Upon review, the treatment team refuted the diagnosis of sarcoidosis but continued treatment with prednisone, IVIG, and methotrexate for CVID-associated myelitis, from which her symptoms have stabilized. Here, we discuss CVID-associated neurological complications, its similarities to sarcoidosis, and a literature review with treatment regimens and outcomes.

## 1. Introduction

CVID is a primary immunodeficiency characterized by a low level of serum immunoglobulin, impaired antibody response, variable T-cell lymphocyte dysfunction, and increased susceptibility to infections [1]. The CNS manifestations of CVID are not well known. In addition to this, CVID can present with many similarities to sarcoidosis. These similarities include arthralgias and nonnecrotizing granulomatous lung disease termed granulomatous lymphocytic interstitial lung disease (GLILD) [1] in CVID. However, the lesser known similarities of these two diseases, specifically for CVID, are its possible effects on the central nervous system (CNS). A literature search revealed a few case reports of myelitis and neurological complications of CVID [2–5]. In this article, we report a patient who presented with significant neurologic ailments secondary to CVID, its diagnostic challenges, and treatment outcomes.

## 2. Case

A 30-year-old Native American female with a past medical history of celiac disease, vitiligo, alopecia areata, recurrent upper respiratory infections, and immune thrombocytopenic purpura (ITP) developed gradually worsening paresthesia and numbness on the right side of her chest radiating down to her right thigh for 2-month duration.

She had a history of three episodes of ITP starting at age 26 until age 29 years which were treated with several courses of prednisone, intravenous immunoglobulin (IVIG), and four doses of rituximab on two separate occasions. Soon after this, she was also diagnosed with celiac disease and alopecia areata. She admitted to a history of recurrent upper respiratory infections. Her family history was notable for thyroid disease in her mother and sister, celiac disease in her sister, and vitiligo in her sister and father. One year before presentation, she noticed stiffness and pain in her knees, ankles, and hands which persisted since then. She denied having a rash similar to erythema nodosum. Two months before presentation, she began to develop progressively worsening numbness on her right chest wall radiating down to her right lower extremity.

**Figure 1:**
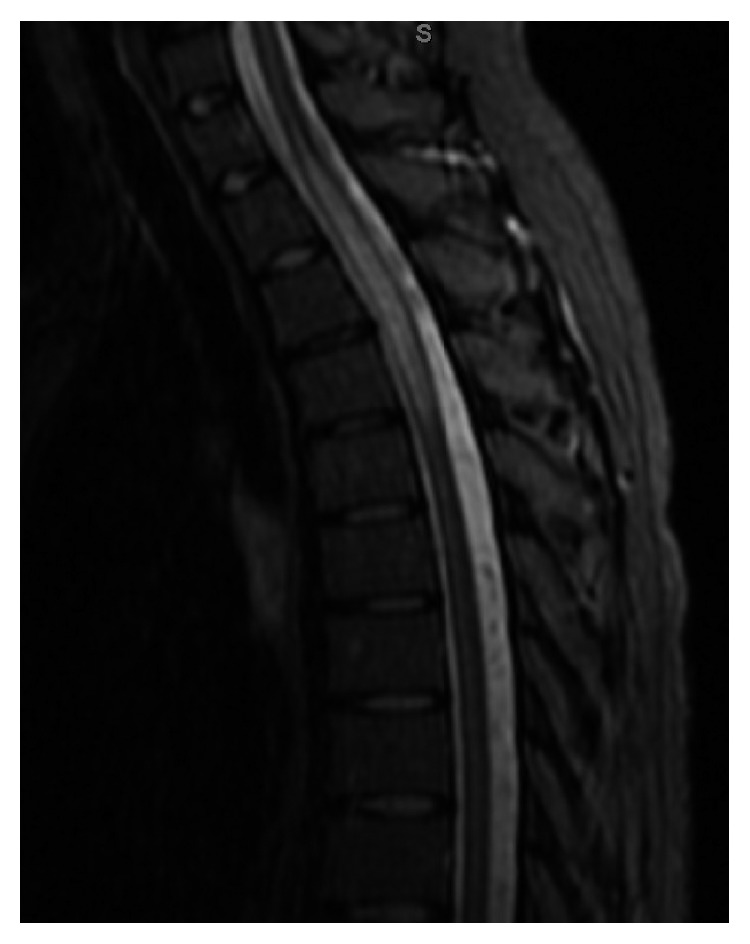
Sagittal view of the thoracic spine showing fluid-sensitive signal hyperintensity from T1 to T5 with associated spinal cord swelling.

**Figure 2:**
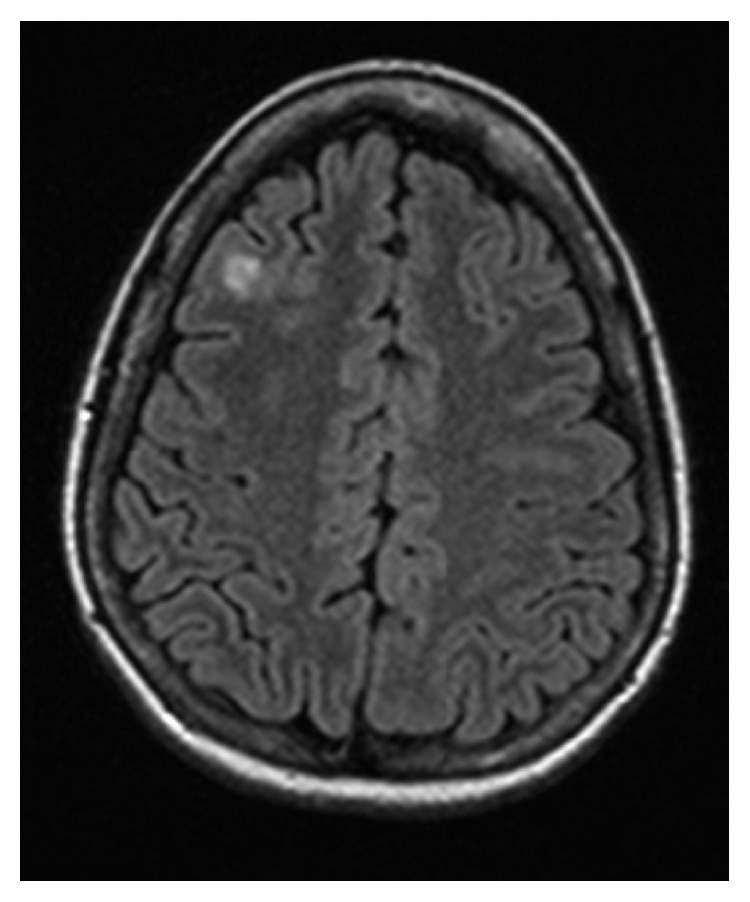
T2 FLAIR signal hyperintensity seen within the right middle frontal gyrus which did not enhance after contrast. There is no adjacent leptomeningeal/dural or parenchymal contrast enhancement.

Physical exam demonstrated loss of sensation on the right side at the level of T7-T8 extending down to the right lower extremity. She did not exhibit any rash. Muscle strength and reflexes were normal in the upper and lower extremities bilaterally. Laboratory data revealed an unremarkable complete blood count (CBC), negative antinuclear antibody (ANA), rheumatoid factor (RF) antibody, Sjogren antibodies, antineutrophil cytoplasmic antibody (ANCA), and anticentromere antibody. Complete metabolic panel was unremarkable except for a low total protein 5.0 g/dL (6.4–8.6 g/dL) and serum globulin of 1.5 g/dL (2.2–4.0 g/dL). Erythrocyte sedimentation rate (ESR) and human immunodeficiency virus (HIV) were unremarkable. Vitamin B12 was borderline low at 267 pg/mL (271–870 pg/mL) with a normal level of methylmalonic acid. MRI of the thoracic spine revealed a fluid-sensitive signal hyperintensity with spinal cord swelling at level T1 through T5 consistent with transverse myelitis (Figure 1); diffuse pulmonary nodules were also found incidentally. MRI of the lumbar and cervical spine was unremarkable. MRI of the brain showed a subcortical FLAIR hyperintensity in the right middle frontal gyrus (Figure 2). Computed tomography (CT) of the chest, abdomen, and pelvis revealed mediastinal lymphadenopathy, as well as diffuse lymphadenopathy in abdomen and pelvis.

Lumbar puncture demonstrated a colorless/clear cerebral spinal fluid (CSF) with normal protein of 25 mg/dL (15–45 mg/dL), normal glucose of 54 mg/dL, normal white blood cell (WBC) of 2 mcL (0–5 mcL), no oligoclonal bands, and normal angiotensin-converting enzyme (ACE) level. CSF immunoglobulin G level was low at 255 mg/dL (768–1632 mg/dL). CSF cytology was negative for malignancy. CSF bacterial, viral, and fungal workup was negative. Serum immunoglobulins revealed a low IgG level of 248 mg/dL (520–1,560 mg/dL), low IgA of 16 mg/dL (88–374 mg/dL), and low IgM of <5 mg/dL (47–206 mg/dL). Anti-NMO antibody was not detected. Due to the chronicity of her symptoms, the decision was made to start the patient on oral dexamethasone 10 mg once every 6 hours.

She underwent endobronchial ultrasound bronchoscopy on the fourth day of admission which examined lymph nodes at two separate locations. Fine-needle aspiration samples obtained using rapid on-site evaluation at these sites identified lymphocytes without pathologic characteristics. Four samples of transbronchial biopsies were obtained which revealed benign respiratory mucosa with mild acute inflammation. Bronchoalveolar lavage (BAL) revealed a WBC of 799/mcL, red blood cell of 19,000/mcL, 57% neutrophils, and 22% lymphocytes. BAL flow cytometry revealed a CD4 : CD8 ratio of 5.8 : 1. She subsequently underwent mediastinoscopy with 5 lymph node excisional biopsies which all revealed reactive hyperplasia, negative for malignancy and granuloma. BAL samples for viral, fungal, and bacterial workup were all negative, including acid-fast bacilli.

**Figure 3:**
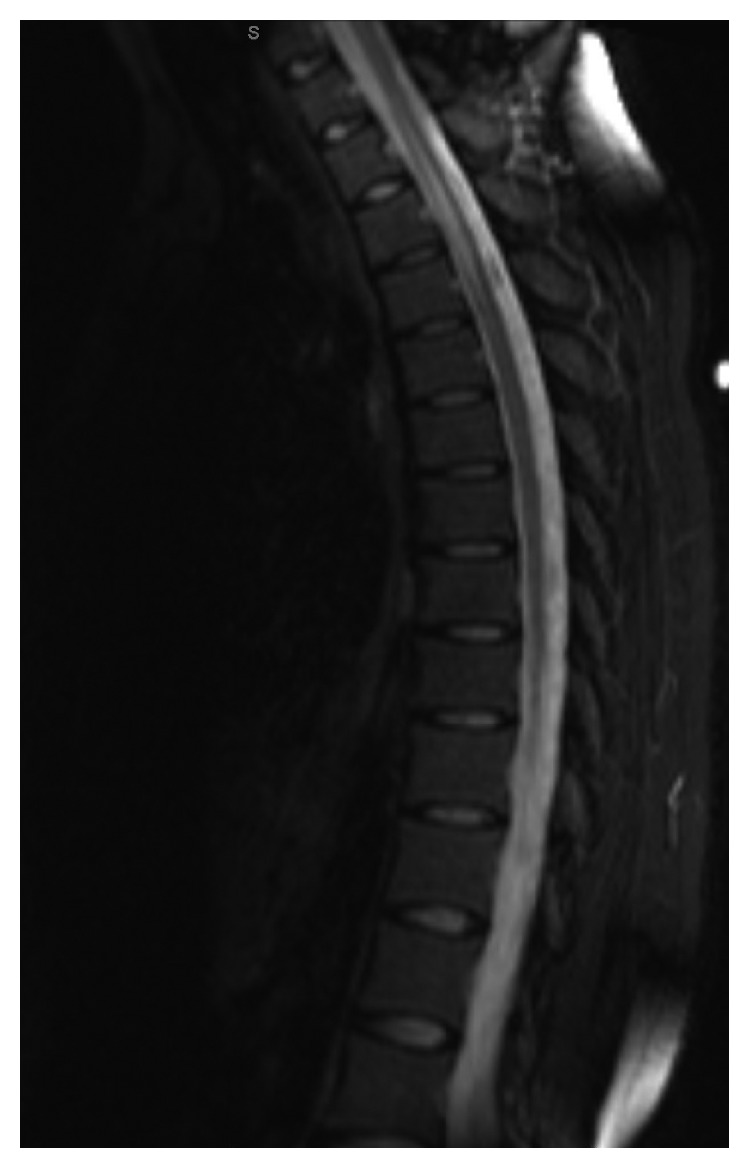
Sagittal view of the thoracic spine demonstrating overall decreased fluid-sensitive hyperintensity within the area of interest from T1 to T5 and improvement of cord swelling.

The patient was diagnosed with CVID-associated myelitis. She was started on treatment with oral dexamethasone 10 mg once every 6 hours for 2 weeks which was then weaned to prednisone 30 mg once a day. She was also started on monthly IVIG replacement which has been continued to the current day. Her two-month follow-up MRI of the thoracic spine did show some improvement in the hyperintensity signal and spinal cord swelling (Figure 3); MRI of the brain showed resolution of the T2 FLAIR hyperintensity in the right frontal gyrus. Her arthralgias resolved, and the numbness on the right side of the body improved but persisted. Due to her persistent numbness, elevated CD4 : CD8 ratio from BAL, and mediastinal lymphadenopathy, an outside physician started her on methotrexate 20 mg intramuscular injection once a week due to their concern for possible sarcoidosis. Of note, the pulmonary function test and electromyography tests were unremarkable. She was treated for CVID and questionable sarcoidosis with prednisone, IVIG, and methotrexate for two years with stability of her symptoms. Her IgG level improved to 1,290 mg/dL (520–1,560 mg/dL), IgA was still low at 9 mg/dL (88–374 mg/dL), and IgM was also still low at 46 mg/dL (47–206 mg/dL); her CD19 was 84 mil/L (44–683 mil/L).

After two years of treatment, an attempt was made to wean her off methotrexate, but this caused an exacerbation in her right lower extremity numbness and arthralgias. MRI of the spine showed a stable thoracic hyperintensity signal without enhancement. MRI of the brain was unremarkable. Repeat CT of the chest showed multiple new ground glass nodules in a central middle to lower lung distribution with stable mild thoracic lymphadenopathy compared to previous images. Repeat CSF studies were again nondiagnostic, and no infectious etiology was found. She was treated with 1 g IV methylprednisolone per day for 3 days during hospitalization which improved her arthralgias but did not improve her numbness. Upon reevaluation of her case, the treatment team refuted the diagnosis of sarcoidosis and concluded that her symptoms were most likely secondary to CVID myelitis, for which she was discharged with prednisone 40 mg once a day and methotrexate 20 mg intramuscular injection once a week, in addition to her monthly IVIG. On two-month follow-up with this treatment regimen, her numbness remains stable but still feels worse than how it was prior to her exacerbation.

## 3. Literature Review

A literature search on PubMed, Web of Science, Cochrane, and Google Scholar databases revealed 4 case reports on the association of CVID and neurological disease [2–5], as well as 13 other cases reporting on some form of “myelitis” in CVID.

In all four (100%) of the cases described, the patients were female with a mean age of 53.25 years (range 40 to 68) which indicates that females may be at increased risk. Our case is unique in that our patient was a younger female at 30 years of age. Unfortunately, ethnicity is mentioned in only one case (Caucasian), so it is unclear how, or if, this factor plays a role.

Our literature search revealed a wide range of reported neurological deficits with CVID. All patients had brisk lower limb reflexes with extensor plantar reflex. 75% of patients had weakness in lower extremities. 50% of patients had a neurogenic bladder, weakness in hands, and/or cerebellar manifestations (ataxia, dysmetria, and poor coordination). It is unclear how these signs could lead to an earlier or easier recognition of this condition, especially as they are quite nonspecific and because there were many other manifestations reported in one patient only. Other findings reported were headaches, neuropathic pain, paresthesias in upper and lower extremities, tremors, loss of vibration and position sense, decreased perception of pain and temperature, spastic gait, visual disturbances, and central scotoma.

In terms of diagnostic tools, all patients had a nonspecific CSF examination (including Gram stain and test for viruses, fungi, and parasites). 75% of patients had a mild protein elevation and 75% of them also had a mild mononuclear/lymphocytic pleocytosis. All patients had evidence of hypogammaglobulinemia. All four case reports indicated the usage of MRI as part of the diagnostic protocol and all demonstrated enhancing lesions in either the spine or the brain. 75% of cases demonstrated lesions at different spinal levels ranging from C-3 to the conus medullaris (one case did not obtain spinal MRI images at the time of discovered brain MRI abnormalities).

In one case, there was involvement of leptomeninges and posteroinferior cerebellar lobe; another patient had a lesion in the right basal forebrain. All of the lesions described had enhancement with gadolinium. This demonstrates the importance of having CVID-associated CNS disease as a differential in patients with similar characteristics. Our patient had CNS involvement with MRI abnormalities from T1 to T5 and the right middle frontal gyrus.

One patient did undergo visual and peripheral nerve evoked potentials (median nerve) which was unremarkable. Other reported infectious pertinent negatives were enterovirus detection by polymerase chain reaction (PCR), varicella-zoster virus PCR, venereal disease research laboratory, Epstein–Barr virus, cytomegalovirus, human immunodeficiency virus, lyme serology, tuberculosis interferon-gamma release assay, Brucella, Trichinella, hydatid, and Toxoplasma immunostaining. In all cases, once the findings described above were obtained, and after some deliberation, a diagnosis of CVID-associated neurological disease was made. It took an average of 2.5 years to attribute it to CVID and an average of 5.75 years to reach it since symptoms started.

In regard to treatment, morbidity, and mortality, all patients received high-dose intravenous glucocorticoids, specifically, methylprednisolone at a dose of 1 g initially for 3–5 days, as well as an IVIG treatment regimen. Three out of the four studies were treated with a glucocorticoid taper that lasted an average of 4.24 months (3 to 6). None of the patients died. We illustrate each case separately and describe MRI findings, treatments, and outcomes in Table 1.

- The first case [2] reported a patient who received three-week interval treatments of IVIG at a dose of 0.5 g/kg, as well as intense inpatient rehabilitation (this is the only case that mentions the use of rehabilitation). She did not receive a glucocorticoid taper. After her stay in inpatient rehabilitation, her neurological symptoms fully recovered. Repeat MRI of the spine showed less intrinsic cord signal change without focal enhancement.
- In the second case [3], in addition to a 6-month glucocorticoid taper, the patient received monthly infliximab at a dose of 5 mg/kg for 9 months, and a repeat MRI showed improvement of her lesions, lymphadenopathy, fevers, and night sweats; however, her neurological symptoms persisted. She was reported to be alive 18 months after diagnosis.
- In the third case [4], this patient received immunoglobulin in different modalities. She did not receive a glucocorticoid taper. The patient first received IVIG 2 g/kg on two consecutive days per month for three months which was then changed to subcutaneous immunoglobulin injections weekly at a dose of 0.2 g/kg for three years. However, her neurological symptoms persisted (brisk lower limb reflexes, spastic gait, decreased perception of pain and temperature, and neurogenic bladder). She did not have any further exacerbations (after having three episodes before treatment). No new lesions were detected on follow-up MRIs.
- The fourth case [5] had a complex medical treatment of firstly being treated for CVID with immunoglobulins, mycophenolate mofetil for lymphocytic interstitial pneumonia, and adalimumab for lymphocytic colitis, while on these treatments, the patient developed neurological symptoms including weakness and ataxia, with two enhancing lesions found in the left cerebellum. Adalimumab was discontinued. After being treated with high-dose glucocorticoids for three days, her neurological symptoms drastically improved as did her repeat brain MRI obtained shortly after. Two months later however, brain MRI revealed an expansile lesion in the right cerebellar lobe. The clinicians then decided to add rituximab to her regimen. Repeat MRI after four doses (650 mg once weekly) demonstrated interval resolution on her lesions (cerebellar and leptomeningeal) without any new areas of enhancement. At the time of the report, she had undergone 3 cycles of rituximab achieving near-complete resolution of her neurological symptoms.

## 4. Discussion

In the setting of transverse myelitis and mediastinal lymphadenopathy, a differential diagnosis of sarcoidosis is appropriate; however, as this case illustrates, CVID should also be considered. The differentiation between CVID and sarcoidosis is sometimes difficult to distinguish, and many can be misdiagnosed as having sarcoidosis for this reason.

Neurological disease in CVID is rare, yet this case and others [2–5] affirm this association. The pathophysiologic process of how CVID affects the CNS is unclear. Transverse myelitis can be associated with infectious, paraneoplastic, neurosarcoidosis, and systemic inflammatory autoimmune conditions including vasculitis, multiple sclerosis, and neuromyelitis optica [6]. Our patient's CSF was peculiar in that it was unremarkable for an infectious, malignant, and inflammatory etiology with normal protein, glucose, and WBC levels, but did show a decrease in IgG level. There have been several postulations of how CVID affects the CNS including an autoimmune injury to the neuronal tissue [7], an unknown infectious etiology [7], or vitamin E deficiency [8], though none of which has been proven yet. Our patient had no infectious etiology found; in addition, she had no evidence of multiple sclerosis or an autoimmune inflammatory process.

Although their clinical manifestations can be very similar, the key difference between CVID and sarcoidosis is the low immunoglobulin levels in CVID and the normal or high immunoglobulin levels in sarcoidosis. In addition, for the diagnosis of sarcoidosis to be made, a confirming biopsy is needed, which our patient did not have despite an adequate number of different samples taken at different locations. Of note, we did not have record of our patient's immunoglobulin levels prior to her first rituximab dose for ITP, which is a known cause of secondary hypogammaglobulinemia [9]. However, her history of vitiligo, recurrent episodes of ITP, and recurrent upper respiratory infections starting at a young age are more supportive of the diagnosis of primary CVID and not secondary hypogammaglobulinemia as an adverse reaction from rituximab use. As supported by another study [10], it is also likely that the rituximab further aggravated her preexisting CVID since she then developed celiac disease and alopecia areata soon afterwards.

As more awareness is made of the association between CVID and its neurological ailments, more studies are needed to establish optimal treatment options. Our literature review demonstrated some benefit with rituximab and infliximab with improvement in MRI findings; however, their neurological symptoms persisted. Danieli et al. illustrate a stable 3-year treatment period without symptomatic exacerbations and improvement in IgG levels with high-dose subcutaneous immunoglobulin without glucocorticoids [4]; however, their patient's neurological symptoms also persisted. Only one patient of the four cases achieved full resolution of symptoms after treatment with high-dose glucocorticoids and IVIG [2]. These variable outcomes demonstrate an unclear optimal treatment regimen for this disease.

Our patient was first treated for CVID-associated myelitis with monthly IVIG and dexamethasone, which was weaned to chronic prednisone. She was not initially treated with high-dose glucocorticoids given the chronicity of her symptoms for two months. The methotrexate was then added on by an outside physician due to their concern for potential sarcoidosis given her CD4 : CD8 ratio, mediastinal lymphadenopathy, and continued numbness despite treatment with IVIG and glucocorticoids. However, it is less likely that our patient had sarcoidosis given the adequate number of negative biopsy samples, normal pulmonary function test, and as further supported by the repeat chest CT findings demonstrating predominant lymphadenopathy in the middle and lower lobes of the lung instead of the upper lobes. Additionally, there have been conflicting results on the usefulness and diagnostic accuracy of BAL CD4/CD8 ratio. A meta-analysis of BAL CD4/CD8 ratio for sarcoidosis found a sensitivity of 70% and specificity of 83% [11], showing that though it may assist in the diagnosis of sarcoidosis, it is not a convincing diagnostic test. Furthermore, this meta-analysis admitted to many different possible biases.

For our patient, in addition to IVIG and chronic prednisone, the methotrexate was continued for CVID-associated myelitis given the patient's stability on this treatment regimen and lack of published data on other treatment regimens. The difficult question that remains is how/when to wean her medication regimen to prevent another exacerbation of symptoms, if possible.

This is the first case reporting the effect of methotrexate as a treatment for CVID-associated neurological disease. One supporting argument to the benefit of methotrexate is that our patient had an exacerbation of her numbness and arthralgias while she was being weaned off methotrexate. After a short course of high-dose glucocorticoids followed by prednisone 30 mg once daily, monthly IVIG, and restarting her previous dose of methotrexate 20 mg intramuscular injection once weekly, her arthralgias improved but her numbness did not. In addition, repeat MRI of the spine showed stable and unchanged findings. It should be noted that the treatment regimen of methotrexate, chronic glucocorticoids, and IVIG was not superior to the other cases reported in the literature review as she had persistent numbness, but its outcome was also noninferior. This case illustrates that methotrexate, in addition to chronic glucocorticoids and IVIG, may have some benefits in preventing CVID-associated neurologic exacerbations; however, further studies are needed to analyze its effects and efficacy.

## 5. Conclusion

This case demonstrates the complex neurological manifestations of CVID and its resemblance to sarcoidosis. Through means of a thorough medical history, radiologic findings, serum laboratory tests, especially immunoglobulin levels, and biopsies, the accurate diagnosis of CVID can be made for prompt and accurate diagnosis, as well as treatment. It is unclear as to what medications may help prevent or best treat the neurological symptoms. Methotrexate may be a potential agent to assist in preventing exacerbation of CVID-associated neurological disease; however, more evidence is needed to support its use.

## Figures and Tables

**Table 1:** Case report MRI findings, treatment, and outcome.

Author	MRI findings	Treatment	Outcome
Farshad et al.	Spine: hyperintensity in the thoracic cord	Chronic glucocorticoids without pulse	Improvement in MRI findings
	Brain: hyperintensity within the right middle frontal gyrus	Methotrexate	Persistent neurological symptoms
		IVIG	

Jabbari et al. [2]	Spine: hyperintensity between T2 and conus medullaris with contrast enhancement	Pulse glucocorticoids without a taper/chronic glucocorticoids	Improvement in MRI findings
	Brain: unremarkable	IVIG	Resolution of neurological symptoms
		Inpatient rehabilitation	

Kumar et al. [3]	Spine: hyperintensity in the cervical and thoracic cord with contrast enhancement	Pulse glucocorticoids followed by chronic taper	Improvement in MRI findings
	Brain: contrast enhancement in the right basal forebrain	Infliximab	Persistent neurological symptoms
		IVIG	

Danieli et al. [4]	Spine: hyperintensity in the cervical and thoracic cord with contrast enhancement	Pulse glucocorticoids without a taper/chronic glucocorticoids	No new lesions in MRI after treatment, unclear if resolved
	Brain: unremarkable	IVIG	Persistent neurological symptoms
		Intramuscular immunoglobulin	

Nguyen et al. [5]	Spine: was not obtained	Pulse glucocorticoids without a taper/chronic glucocorticoids	Improvement in MRI findings
	Brain: two enhancing lesions in left cerebellum as well as leptomeningeal enhancement	Rituximab	Improvement/near resolution of neurological symptoms
		IVIG	
